# Neighboring Pd single atoms surpass isolated single atoms for selective hydrodehalogenation catalysis

**DOI:** 10.1038/s41467-021-25526-2

**Published:** 2021-08-30

**Authors:** Chiheng Chu, Dahong Huang, Srishti Gupta, Seunghyun Weon, Junfeng Niu, Eli Stavitski, Christopher Muhich, Jae-Hong Kim

**Affiliations:** 1 Department of Environmental Science, Zhejiang University, Hangzhou, China; 2 Department of Chemical and Environmental Engineering, Yale University, New Haven, CT USA; 3 NSF Nanosystems Engineering Research Center for Nanotechnology Enabled Water Treatment (NEWT), Houston, TX USA; 4 School of Environment and Civil Engineering, Dongguan University of Technology, Dongguan, Guangdong China; 5 School for the Engineering of Matter, Transport, and Energy, Arizona State University, Tempe, AZ USA; 6 School of Health and Environmental Science, Korea University, Seoul, Korea; 7 National Synchrotron Light Source-II, Brookhaven National Laboratory, Upton, NY USA

**Keywords:** Catalyst synthesis, Heterogeneous catalysis, Pollution remediation

## Abstract

Single atom catalysts have been found to exhibit superior selectivity over nanoparticulate catalysts for catalytic reactions such as hydrogenation due to their single-site nature. However, improved selectively is often accompanied by loss of activity and slow kinetics. Here we demonstrate that neighboring Pd single atom catalysts retain the high selectivity merit of sparsely isolated single atom catalysts, while the cooperative interactions between neighboring atoms greatly enhance the activity for hydrogenation of carbon-halogen bonds. Experimental results and computational calculations suggest that neighboring Pd atoms work in synergy to lower the energy of key meta-stable reactions steps, i.e., initial water desorption and final hydrogenated product desorption. The placement of neighboring Pd atoms also contribute to nearly exclusive hydrogenation of carbon-chlorine bond without altering any other bonds in organohalogens. The promising hydrogenation performance achieved by neighboring single atoms sheds light on a new approach for manipulating the activity and selectivity of single atom catalysts that are increasingly studied in multiple applications.

## Abstract (web summary)

Single atom catalysts have exhibited high selectivity for hydrogenation, yet improved selectively is often accompanied by loss of activity. Here the authors report that synergistic interactions of neighboring Pd single atoms lead to both high activity and selectivity for hydrodehalogenation catalysis.

## Introduction

The essential role of heterogeneous catalysis in chemical synthesis, fuel production, and pollutant remediation is driving extensive research efforts in optimizing reactivity and selectivity of catalyst materials. Reducing particle size, often down to nanoscale, and thereby increasing exposure of surface metal atoms have been a common, effective strategy to improve their performance and atom-utilization efficiency^1^. Single atom catalysts (SACs) represent a rapidly emerging trend in catalyst design, where atomic dispersion ensures that each metal atom is exposed and available for catalytic reactions^1,2^. The resultant reduced material usage is particularly attractive for noble metal catalysts that are highly effective in reductive reactions (e.g., hydrogenation) but cost-prohibitive at large scales.

Recent studies suggest that the single-site nature empowers SACs’ superior selectivity in select catalytic schemes. Uniform geometric and electronic structure of the catalytic center can favor only one specific adsorption pattern and reaction pathway, resembling that of homogeneous metal catalysts (e.g., ligand-bound aqueous phase metal complexes)^3^. This characteristic of SACs is highly desirable in hydrogenation processes that often require selective reduction of the target functional group while keeping the others intact^3^. For instance, highly selective hydrogenation of alkyne^4,5^, diene^6,7^, styrene^8^, nitroarene^9^, and benzaldehyde^10^ has been realized through engineering the geometric configuration of a single metal site that tunes the adsorptivity of reactants/intermediates^3^. However, the high hydrogenation selectivity of SACs is often accompanied by the loss of activity^3,4,6,11^, since the adsorption of a target molecule on a single metal site sterically hindered the adsorption and dissociation of the key reactant, i.e., H_2_^6^.

This begs an important question: Can we improve the hydrogenation activity of SACs while maintaining its high selectivity? One potential strategy is to shorten the distance between single atoms such that two neighboring single atoms provide adjacent active sites; this architecture partially resembles nanocatalysts but still maintains the benefit of single atom dispersion. For example, it was recently reported that neighboring Fe single atoms efficiently cleave O-O bonds, a key step in realizing the preferred four-electron oxygen reduction reactions in fuel cells^12^. Because neighboring SACs resemble sparsely isolated SACs (i-SACs) in electronic and geometric structures, they likely retain the catalytic selectivity of i-SACs, while providing higher activity. Nevertheless, there is a dearth of information on which catalytic reactions can benefit from neighboring single atom configurations and a significant knowledge gap in understanding their mechanistic functions.

Here we report the synergistic interactions of neighboring Pd single atoms that contribute to a superior performance for selective hydrogenation of organohalides. Organohalides occur widely as agrochemicals, pharmaceuticals, solvents, and surfactants, as well as toxic by-products in the effluent of hydraulic fracturing^13,14^. Concerns over the adverse effects of organohalides on the ecosystem and human health have led to rigorous regulations and research needs for developing efficient dehalogenation techniques^13^. We demonstrate that a neighboring Pd SAC (n-Pd_1_) exhibits both high activity and selectivity for hydrodehalogenation catalysis, significantly surpassing both isolated Pd SAC (i-Pd_1_) and Pd nanoparticles (Pd_nano_). We discuss, based on density functional theory (DFT) calculations, why neighboring single atom configuration is essential for enhanced kinetics and selectivity.

## Results and discussion

### Synthesis and structural characterization of Pd SACs

**Fig. 1:**
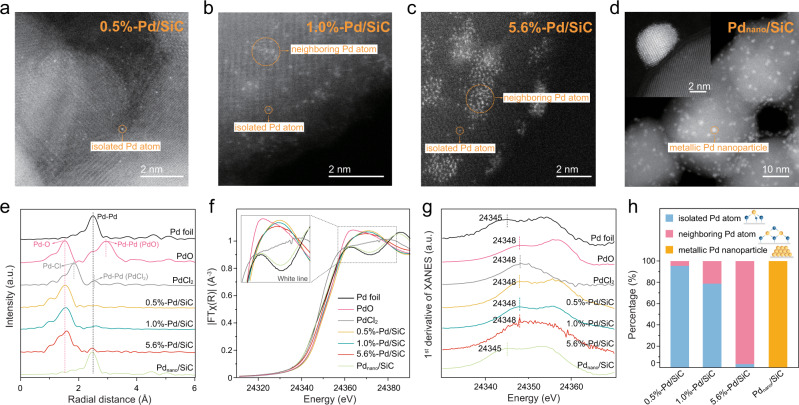
Structural characterizations of Pd/SiC. **a**–**d** High-angle annular dark-field scanning transmission electron microscopy (HAADF-STEM) images of 0.5%-Pd/SiC, 1.0%-Pd/SiC, 5.6%-Pd/SiC, and Pd_nano_/SiC (the inset shows the crystal structure of Pd nanoparticle). **e** Pd *K*-edge EXAFS spectra in *R* space for Pd/SiC and references. **f**–**g** Pd *K*-edge normalized XANES spectra and first derivative curves of Pd/SiC and references. **h** Compositions of isolated Pd atom, neighboring Pd atom, and metallic Pd nanoparticle.

We synthesized Pd SAC by first binding Pd precursors (PdCl_4_^2−^) onto the amine-modified SiC surface via electrostatic attraction and subsequently reducing the surface-anchored precursors by a moderate ultraviolet C (UV-C) irradiation (7.8 mW cm^−2^)^10,15^. Energy-dispersive X-ray spectroscopy suggests successful loading of Pd atoms that are uniformly distributed across the SiC surface (Supplementary Fig. 1). X-ray photoelectron spectroscopy (XPS) elemental analysis (Supplementary Fig. 2) shows that the binding energy of Pd 3d_5/2_ is close to that of PdCl_2_ reference before UV-C irradiation (338.0 eV) and that of PdO reference after UV-C irradiation (335.9 eV). The atomic dispersion of Pd and the absence of Pd nanoparticles are confirmed by high-angle annular dark-field scanning transmission electron microscopy (HAADF-STEM) images, where the radius of Pd species is estimated to be ~1.5 Å (Fig. 1a–c). Figure 1a shows that Pd atoms in 0.5%-Pd/SiC (Pd mass loading = 0.5%) are sparsely distributed as i-Pd_1_. As Pd loading increases to 1.0%, a small fraction of Pd atoms form groups of neighboring atoms, n-Pd_1_ (Fig. 1b), while most remain as i-Pd_1_. At 5.6%, most Pd atoms form groups (Fig. 1c) within which neighboring Pd atoms are randomly distributed. This n-Pd_1_ distribution pattern is in marked contrast to Pd clusters (Pd_nano_) that show a much higher Pd atom density and an ordered crystal structure with clear lattice fringes (Fig. 1d).

The atomic dispersion of Pd in 0.5%-Pd/SiC, 1.0%-Pd/SiC, and 5.6%-Pd/SiC was confirmed by the absence of Pd-Pd coordination from Fourier-transformed extended X-ray absorption fine structure spectroscopy (FT-EXAFS) analysis at the Pd *K*-edge (Fig. 1e). Comparison of FT-EXAFS spectra with references indicate that Pd is coordinated with Cl atoms before UV-C irradiation (Supplementary Fig. 3) and coordinated with O atoms after UV-C irradiation (Fig. 1e). The distinct Pd-O coordination is in stark contrast with the metal-like Pd-Pd coordination observed in Pd_nano_/SiC (Fig. 1e). Best-fit parameters extracted from the Pd *K*-edge FT-EXAFS spectra suggest that 0.5%-Pd/SiC, 1.0%-Pd/SiC, and 5.6%-Pd/SiC share a high level of similarity in the first shell structure of Pd single atoms (Supplementary Fig. 4), with an average Pd-O coordination number of 3.2–3.4 and an interatomic distance of 2.03–2.05 nm (Supplementary Table 1). Notably, the second shell Pd-Pd coordination (i.e., peak at 2.4 Å in FT-EXAFS) of a PdO standard is not observed in 0.5%-Pd/SiC and 1.0%-Pd/SiC (Fig. 1e). In comparison, a small peak at 2.4 Å, corresponding to the second shell Pd-Pd coordination, is observed in 5.6%-Pd/SiC, confirming the formation of Pd-O-Pd structure in neighboring Pd single atoms. We note that the FT-EXAFS pattern of 5.6%-Pd/SiC is distinct from those of Pd clusters, which exhibit a prominent Pd-Pd coordination with high coordination number (e.g., 6.3–11.0 with Pd cluster sizes of 1.3–10.5 nm)^16^.

X-ray absorption near edge structure (XANES) analysis shows that the white line (WL) intensity of Pd single atoms is between that of Pd^0^ and Pd^2+^ (Fig. 1f), suggesting that Pd atoms are positively charged with partially unoccupied 4d orbitals. The first peak of the XANES derivative of 0.5%-Pd/SiC, 1.0%-Pd/SiC, and 5.6%-Pd/SiC (i.e., peak at 24,348 eV; Fig. 1g) matches that of PdO and PdCl_2_ references. This indicates that the valence state of the Pd single atoms is close to +2, agreeing with the coordination of Pd single atoms with electrophilic O atoms observed by FT-EXAFS spectra (Fig. 1e). The stable Pd oxidation state with increasing loading amount also exclude the formation of Pd clusters, which, if occurs, should shift Pd oxidation state toward lower value. A significantly higher binding energy of the Pd 3d_5/2_ peak (335.9 eV) compared to that of metallic Pd (335.3 eV) in the XPS spectrum also confirmed the presence of oxidized Pd atoms (Supplementary Fig. 5). DFT calculations confirm that Pd atoms are positively charged, with Bader charges of +0.8 and +0.9 for single atom Pd and PdO reference, respectively. In contrast, the oxidation state of Pd atoms in Pd_nano_/SiC is close to that of Pd foil, as suggested by similar WL intensity (Fig. 1f) and first derivate XANES (first peak at 24,345 eV; Fig. 1g).

The above results together suggest that Pd in 0.5%-Pd/SiC, 1.0%-Pd/SiC, and 5.6%-Pd/SiC are all in Pd_1_ state (coordinated with O atoms and hence positively charged). We define Pd atoms without a secondary Pd atom in the second shell coordination (i.e., 3.0 and 3.4 Å in PdO standard)^17^ as i-Pd_1_; and Pd atoms interacted with one or more Pd atoms in the second shell coordination as n-Pd_1_. Based on HAADF-STEM images, we estimate that Pd atoms in 0.5%-Pd/SiC are mostly i-Pd_1_ (95.2%, 40 isolated and 2 neighboring Pd atoms), whereas 1.0%-Pd/SiC consists of both isolated (80.4%, 41 isolated and 10 neighboring Pd atoms) and neighboring Pd atoms (19.6%) (Fig. 1h). Pd atoms in 5.6%-Pd/SiC are dominantly n-Pd_1_ (96.9%, 10 isolated and 313 neighboring Pd atoms).

### Hydrodehalogenation activity and selectivity

**Fig. 2:**
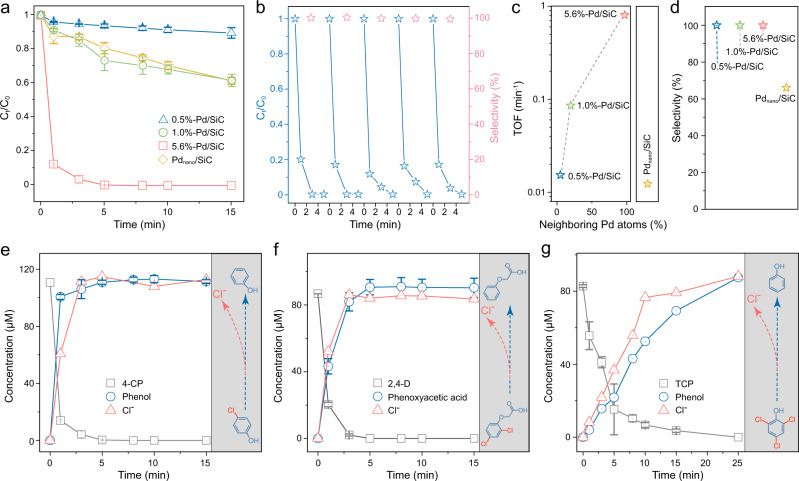
Catalytic performance of Pd/SiC in the hydrodehalogenation of organohalides. **a** Hydrodehalogenation kinetics of 4-CP by Pd/SiC. **b** Conversion ratio and selectivity of 4-CP at 5 min by 5.6%-Pd/SiC over five consecutive cycles. **c** TOF per Pd atom basis calculated with the initial zero-order rate constant of 4-CP hydrogenation. **d** Selectivity of hydrodehalogenation. **e**–**g** Kinetic plots of the organohalide hydrogenation and product generation. The Cl^−^ concentrations in **f** and **g** were divided by a factor of two (i.e., each 2,4-D molecule contains two Cl atoms) or three (i.e., each TCP molecule contains three Cl atoms) to visualize the comparisons. Experimental conditions: catalyst (0.5 g L^−1^), H_2_ (1 atm), room temperature (20 °C). Error bars represent standard deviations from triplicate experiments.

We tested the aqueous suspension of Pd/SiC for catalytic hydrodehalogenation of 4-chlorophenol (4-CP, a model organohalogen compound) under H_2_ purging at room temperature. To the best of our knowledge, this is the first report where SACs were used to catalyze H_2_-driven dehalogenation reaction. All Pd/SiC materials degraded 4-CP, but they differed in kinetics: 5.6%-Pd/SiC > Pd_nano_/SiC >> 1.0%-Pd/SiC > 0.5%-Pd/SiC (Fig. 2a). The kinetics achieved by 5.6%-Pd/SiC was extremely fast, achieving over 80% of 4-CP decay within 1 min. The pseudo-first-order rate constant was 2.1 min^−1^, which is the fastest kinetics ever reported for catalytic 4-CP removal (Supplementary Table 2). Control experiment shows negligible 4-CP degradation on bare SiC (Supplementary Fig. 6) or by 5.6%-Pd/SiC without H_2_ purging (Supplementary Fig. 7), confirming the reaction on Pd catalytic centers as the only pathway for 4-CP removal. No significant decreases in the activity of Pd SACs were observed over a 5-cycle of repetitive use (Fig. 2b). XPS (Fig. S5), XANES (Supplementary Fig. 8a), and EXAFS (Supplementary Fig. 8b) analyses show no significant change in chemical composition of 5.6%-Pd/SiC after 1-week hydrogenation. We attribute the durability of atomically dispersed Pd to the strong coordination with O atoms.

Figure 2c shows that turnover frequency (TOF; per Pd atom basis) of 5.6%-Pd/SiC is 65 times higher than Pd_nano_/SiC. This comparison (Pd_1_ versus Pd_nano_) highlights the advantage of atomic dispersion, which maximizes the exposure of each Pd atom as a catalytic center. More interestingly, the TOF dramatically increased at higher Pd loadings: 5.6%-Pd/SiC > 1.0%-Pd/SiC > 0.5%-Pd/SiC. A positive correlation between neighboring Pd atom composition and TOF (Fig. 2c) suggests that the activity of n-Pd_1_ is much higher than that of i-Pd_1_. This would not have resulted if all Pd_1_ atoms catalyzed the reaction at equal efficiency. The result indicates that Pd single atom can more efficiently catalyze the reaction when it has other Pd atoms in its vicinity.

We found that the removal of 4-CP by Pd/SiC almost exclusively proceeded through hydrodehalogenation. Results of product analysis (Fig. 2e) suggest that >99% selectivity toward dehalogenation (i.e., >99% of 4-CP converted to phenol with concomitant release of Cl^−^) was achieved by 5.6%-Pd/SiC. All Pd SACs are highly selective toward cleavage of the C-Cl bond, with a dehalogenation selectivity >99% (Fig. 2d). No significant decrease in 4-CP dehalogenation selectivity was observed within 5 cycles (Fig. 2b). In contrast, much lower dehalogenation selectivity (66%) was observed in Pd_nano_/SiC (Fig. 2d), consistent with previous claims that metallic Pd nanoparticles are non-selective between, for example, hydrogenation of conjugated carbons in a benzene ring versus carbons in C-Cl bonds^18,19^.

### Mechanisms of neighboring Pd single atom catalysis

To understand the atomic-level behavior of i-Pd_1_, n-Pd_1_, and Pd_nano_, we used DFT to calculate the 4-CP hydrodehalogenation reaction pathways. In this work, we only calculate the meta-stable states along the path. The energy pathways on the various surfaces outlined by the meta-stable state deviate from each other by >1 eV, and the endothermic steps are desorption step, which are normally barrierless. Therefore, the highest reaction barriers that are accessible at experimental conditions would not change the conclusions reached here, and we leave detailed transition state analysis to future work.

**Fig. 3:**
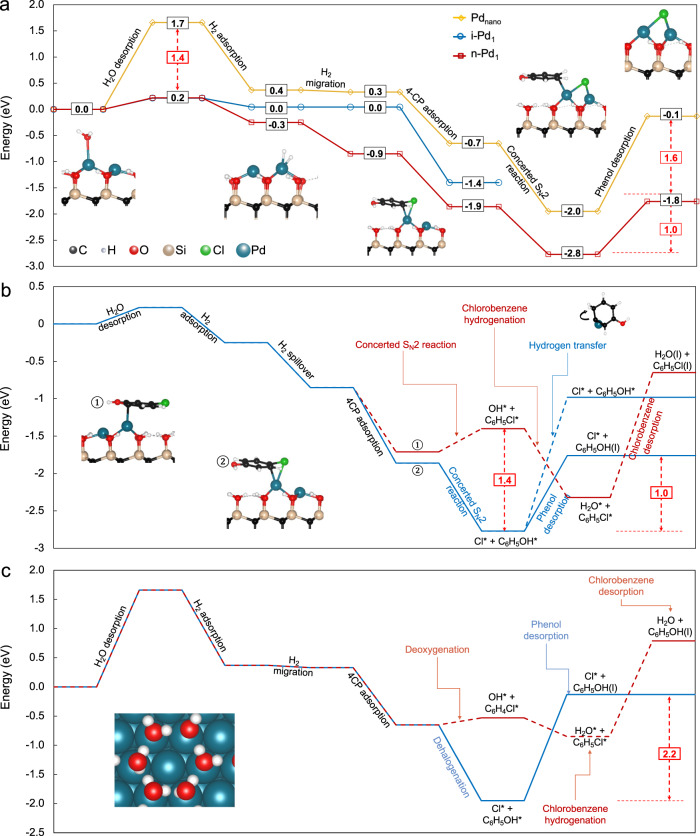
DFT-calculated minimum energy pathway of 4-CP hydrogenation. **a** Hydrodehalogenation on i-Pd_1_, n-Pd_1_, and Pd_nano_. All steps are exothermic except for H_2_O, phenol, and Cl^−^ desorption (not calculated here). **b** Alternative 4-CP hydrogenation pathways on n-Pd_1_ (i.e., C-O bond cleavage and benzene ring hydrogenation). **c** Alternative 4-CP hydrogenation pathways on Pd_nano_. The blue, black, red, tan, and white spheres in geometrical models are Pd, C, O, Si, and H atoms, respectively. The solid and dashed lines represent minimum energy path and other reaction pathways, respectively.

DFT calculations predict that at least two neighboring Pd atoms are necessary for C-Cl bond cleavage, thus explaining the absence of hydrodehalogenation activity on i-Pd_1_. On n-Pd_1_, the cleaved Cl^−^ migrates to the neighboring Pd atom, stabilizing the anion and providing space for H to attack the Pd bound C (Fig. 3a). This does not occur on i-Pd_1_ because Cl does not readily dissociate from the C. In all attempts to force the Cl away from the C onto the i-Pd_1_ or the SiC surface, the Cl relaxed back to the phenol. Therefore, several H attack routes from the i-Pd_1_ and SiC surface were tested with the C-Cl bond intact; in all cases the H relaxed back onto the SiC surface, and the C-Cl bond remained intact. Hence, hydrodehalogenation reaction on i-Pd_1_ is prohibited by steric hindrance and absence of a second metal site for Cl adsorption prevents further reaction.

We screened several possible reaction paths on the n-Pd_1_ and Pd_nano_ structures. The lowest energy path proceeds as follows: (1) H_2_O desorption from the Pd, (2) H_2_ adsorption on Pd, (3) H_2_ dissociation, (3) H spillover onto the SiC or Pd surface, (4) 4-CP adsorption, (5) concerted S_N_2-like C-Cl bond cleavage and C-H bond formation, and (6) phenol desorption. This reaction path is shown in Fig. 3a. The final Cl^−^ desorption was not included in the analysis as it forms a highly solvated solution anion. We predict that phenol desorption is the rate limiting step on n-Pd_1_ (1.01 eV) and on Pd_nano_ (1.82 eV), as shown in Fig. 3a. The weaker binding of adsorbates on n-Pd_1_ explains its efficient hydrodehalogenation compared to Pd_nano_.

The lower barriers for desorption from n-Pd_1_ as compared to Pd_nano_ stems from weaker individual bond strengths and fewer bonds, which much be broken. The removal of one H_2_O molecule (1.02 eV) from Pd_nano_ is more endothermic than on n-Pd_1_ (0.22 eV) because the hexagonal H-bonding network of water on the Pd_nano_ surface^20^ stabilizes H_2_O adsorption there while only one water can adsorb on n-Pd_1_. This is further exacerbated because three H_2_O molecules must desorb from Pd_nano_ to enable 4-CP adsorption, resulting in the 1.66 eV water desorption barrier. Likewise, the energy barrier for phenol desorption from n-Pd_1_ (1.01 eV) is significantly lower than from the Pd_nano_ surface (1.82 eV), because only one C-Pd bond breaks instead of six. Overall, the lower energy of phenol and solvent desorption from n-Pd_1_ as compared to Pd_nano_, results in higher hydrodehalogenation activity of SACs.

We also calculated possible competing reaction paths to 4-CP hydrodehalogenation on n-Pd_1_ and Pd_nano_ to understand the high selectivity of n-Pd_1_. The alternate mechanisms include hydrogenation of the C-O bond (hydrodeoxygenation) and hydrogenation of the conjugated carbons in the benzene ring (ring hydrogenation). On n-Pd_1_, C-O bond cleavage is 1.37 eV higher in energy than C-Cl bond cleavage (Fig. 3b). This corresponds to C-O bond cleavage, and thus hydrodeoxygenation, being 10^−22^ times less likely to occur than C-Cl bond cleavage (step 5) at room temperature based on a Boltzmann distribution. This prevents the hydrogenation of 4-CP proceeding along the deoxygenation path to chlorobenzene  (step 6*).

The first step in benzene ring hydrogenation on n-Pd_1_ is the migration of a H from the C anchored to the Pd to its neighboring C. This step is endothermic by 1.79 eV, which is significantly higher in energy than phenol desorption (1.01 eV). In other words, dissociation of the C-Cl bond and hydrogenation of C on n-Pd_1_ is followed by phenol desorption, thus preventing further hydrogenation of the benzene ring. This is in contrast to Pd_nano_ where all C atoms interact with the Pd surface and are activated, as indicated by the 1.45 Å mean C-C bond length (compared to 1.40 Å, mean benzene ring bond length; Fig. 3c), meaning that multiple side reactions are possible. These calculations confirm our experimental observation that n-Pd_1_ rapidly and selectively cleaves the C-Cl bond producing a dehalogenated molecule.

### Selective hydrodehalogenation for environmental remediation

While the unprecedented selectivity of n-Pd_1_ toward breaking C-Cl bonds of organohalogens can be exploited for various industrial chemical processes, one application of potentially significant impact is hydrodehalogenation of halogenated contaminants of significant public health concerns, such as 2,4-dichlorophenoxyacetic acid (2,4-D; a widely used herbicide and possible human carcinogen) and 2,4,6-trichlorophenol (TCP; a pesticide and possible human carcinogen) in addition to 4-CP discussed above^13^. Note that selectively breaking only C-Cl bonds and dehalogenating organics that are polyhalogenated and contain multiple interfering moieties (e.g., ether, ketone) have not been possible with past Pd catalysts. We observed that both 2,4-D and TCP are completely removed in 5 and 15 min, respectively, confirming the high catalytic activity of n-Pd_1_ in 5.6%-Pd/SiC (Fig. 2f, g). The reduction of polyhalogenated 2,4-D and TCP followed a step-by-step dehalogenation, with the emergence and depletion of mono-chlorinated and di-chlorinated intermediates (Supplementary Figs. 9 and 10). The formation and depletion of dehalogenation intermediates were also evidenced by the changes in Cl contained in the intermediates (Supplementary Figs. 11 and 12). Phenoxyacetic acid and phenol comprised >99% of the final hydrogenation products of 2,4-D (Fig. 2f) and TCP (Fig. 2g); consistently, >99% of Cl atoms in 2,4-D (Fig. 2f) and TCP (Fig. 2g) are recovered as Cl^−^, further demonstrating the high selectivity of n-Pd_1_ for breakage of only C-Cl bonds without any side reactions (e.g., hydrogenation of ether, ketone, or benzene moieties in 2,4-D and TCP).

The high hydrodehalogenation selectivity of neighboring Pd single atoms is also critical for the practical environmental remediation scenarios where competing species are present. For instance, we found that 5.6%-Pd/SiC could also rapidly hydrodehalogenate 4-CP in the presence of natural organic matter (5 mg-C L^−1^), a group of naturally occurring aromatic compounds that typically interfere with the hydrogenation processes in a natural water matrix (Supplementary Fig. 13)^21^. The presence of natural organic matter indeed inhibited 4-CP hydrogenation by Pd_nano_/SiC due to dominant competing hydrogenation reactions (e.g., benzene ring hydrogenation; Supplementary Fig. 14).

We demonstrate that neighboring Pd single atoms exhibit unprecedently high activity and selectivity for cleavage of the carbon–halogen bond, which are difficult to achieve by either sparsely dispersed single atoms or metallic Pd nanoparticles. Experimental results and computational calculations suggest that at least two neighboring Pd atoms can synergistically enhance hydrodehalogenation reaction kinetics by lowering the reaction energy of key reactions steps, i.e., initial water desorption and final phenol desorption. The high selectivity of n-Pd_1_ originates from significantly lower reaction energy for hydrodehalogenation than other possible reaction pathways, which is in stark contrast to the undifferentiated H attacks mediated by Pd_nano_. This result highlights that, for the reaction examined in this study and potentially for other reactions, there is plenty of room for catalyst design between isolated single atoms and nanoparticles to achieve higher activity and selectivity. Our findings present a new approach for manipulating the activity and selectivity of SACs that are increasingly studied in a wide range of applications by carefully controlling the metal spatial distribution in nanoscale and sub-nanoscale.

## Methods

### Catalyst synthesis

#### Single atom Pd/SiC

In order to improve the electrostatic adsorption of Pd precursor (i.e., PdCl_4_^2−^), surface modification of SiC was conducted by dispersing 0.5 g SiC in 250 hexane under ultrasonication for 30 min, followed by the addition of 2 mL aminopropyltrimethoxysilane and ultrasonication for another 60 min. We note that the surface amines on SiC also serve to improve catalyst dispersion in water solution during the performance tests (Supplementary Fig. 15). Surface-modified SiC was separated by centrifugation, washed with ethanol and deionized water (twice each), and dried at 80 °C overnight. Following this, 80 mg surface-modified SiC was dispersed in 60 mL deionized water under ultrasonication for 30 min, followed by the addition of 0.38, 0.76, or 4.56 mL H_2_PdCl_4_ (10 mM). The mixture was stirred for 60 min and irradiated with UV-C (7.8 mW cm^−2^) for 8 h at room temperature. As-prepared Pd/SiC was washed with water and ethanol (twice each) and dried in a vacuum oven at 80 °C overnight. The Pd loading amount was determined by inductively coupled plasma mass spectrometry (ICP-MS, PerkinElmer SCIEX Elan DRC-e). Pd atoms did not form direct coordination with amine, as indicated by the unchanged binding energy of Pd in XPS analysis (Supplementary Fig. 16) when amine species were blocked by adding amine capping agent Sulfo-NHS-Acetate (amine-capping evidenced by the dramatic decline in the zeta potential of 5.6%-Pd/SiC; Supplementary Fig. 17).

#### Pd_nano_/SiC

Pd nanoparticles were loaded on SiC through a hydrothermal method by heating suspensions containing 60 mg surface-modified SiC (60 mg), 45.2 µmol H_2_PdCl_4_, and 5% (v/v) isopropanol as reductant at a heating rate of 10 °C min^−1^ to 120 °C and annealing for 4 h. As-prepared Pd_nano_/SiC was separated by centrifugation, washed with water and ethanol (twice each), and dried in a vacuum oven at 80 °C overnight. The Pd loading amount was determined to be 6.2% (w/w) by ICP-MS.

### Catalyst characterization

HAADF-STEM images were taken on a Titan Themis Z STEM (ThermoFisher Scientific, USA) operated at 200 kV, coupled with a probe aberration-corrector to improve imaging spatial resolution to <1 Å. The X-ray absorption spectroscopy spectra at Pd *k*-edge were measured at Beamline 8-ID (ISS) of the National Synchrotron Light Source II at Brookhaven National Laboratory, using a Si (111) double crystal monochromator and a passivated implanted planar silicon fluorescence detector at room temperature, with energy calibrated using Pd foil. The catalyst samples were pressed into a pellet and sealed in Kapton films for XAFS measurements. XAFS data was analyzed using the Athena and Artemis software for conversion of raw data to *µ*(*E*) spectra, background subtraction and normalization, Fourier transformation and plotting, and fitting in *k*-space and *R*-space.

### Evaluation of hydrodehalogenation performance

Hydrodehalogenation of organohalogens was performed in a serum glass bottle sealed with rubber septa at room temperature (20 °C). The suspension containing 0.5 g L^−1^ catalyst was purged with H_2_ (purity = 99.9995%) for 5 min prior to the reaction, followed by addition of aqueous stock (5 mM) of 4-CP (Sigma, 99%), 2,4-D (Sigma, 99%), or TCP (Sigma, 98%) using a syringe to initiate the reaction (initial organohalide concentration was ~100 µM). Aliquots (250 µL) were taken at defined time points from the sample solutions by syringe and immediately purged with air to terminate the reactions. To test the catalyst recyclability, 4-CP was repetitively added up to 5 cycles and aliquots (150 µL) were taken at defined time points in each cycle.

### Quantification of organohalogens and hydrogenation products

Aliquots were centrifuged and 50 µL supernatants was injected into a C18 column at 20 °C, analyzed with an Agilent high-performance liquid chromatography (HPLC) coupled to a photodiode array detector (see Supplementary Table 3 for detailed HPLC methods), and quantified with corresponding analytical standards. For quantification of Cl^−^, the pH of aliquots was adjusted to ~13 by adding NaOH (1 M) to detach Cl^−^ from the positively charged SiC surface. Afterwards, the aliquots were centrifuged and 100 µL supernatants were removed, diluted, and injected into a Dionex ion chromatography, and quantified with corresponding analytical standards.

### Computational methodology

Periodic boundary condition DFT calculations were performed at the Perdew–Burke–Ernzerhof level, as implemented in the Vienna Ab initio Simulation Program (VASP)^22–24^. Non-local van der Waals interactions were accounted for through the DFT-D3 correction, which is based on the method of Grimme et al.^25^. The wave function was formed from a summation of plane waves with energies <500 eV. We used the projected-augmented wave method pseudopotentials^26^ to reduce the number of plane waves required, where we explicitly included the H 1s, O, and C 2s2p, Cl 3s3p, and Pd 5s4d electrons.

Pd and single/neighboring atom Pd catalyst surfaces were repressed by a Pd slab consisting of a supercell with a (111) terminated 4 × 4 × 6 primitive units and a single and neighboring Pd atoms on a (001) terminated (6 × 6 × 4) Si-C slab, respectively. The surface terminations were chosen because they represent the low-energy surfaces^27–29^. Both slabs were separated by at least 12 Å of vacuum space. The Brillion zone was sampled on a 6 × 6 × 1 and 2 × 2 × 1 Γ-point centered Mohnkorst Pack mesh for the Pd and SiC surfaces, respectively. A layer of H_2_O and dissociated H_2_O (OH) covered the Pd surface and SiC surface, respectively, to mimic the water environment from the experiments. The Pd atoms catalysts were bonded to the SiC surface via two surface oxygen (Fig. 1b). All geometries were relaxed to 1.0 × 10^−4^ eV. The energies of molecules in water are calculated with implicit solvent using the VASPsol module^30,31^. Geometric structure representations were generated using VESTA. The activation barriers for the exothermic steps were not calculated, as they are likely to be lower in energy than those for endothermic desorption.

## Supplementary information

Supplementary Information

## Data Availability

The data that support the findings of this study are available from the corresponding authors upon reasonable request.
