# Supplementary material for: Neighboring Pd single atoms surpass isolated single atoms for selective hydrodehalogenation catalysis
Source: Nat Commun. 2021 Aug 30;12:5179. doi: 10.1038/s41467-021-25526-2 (PMC8405729; doi:10.1038/s41467-021-25526-2)
Supplement: Supplementary file 1 — Supplementary Information [file 41467_2021_25526_MOESM1_ESM.pdf]

Supplementary Information

Neighboring Pd Single Atoms Surpass Isolated Single Atoms for Selective Hydrodehalogenation Catalysis

Chiheng Chu,[1,2,3,#] Dahong Huang,[2,3,4,#] Srishti Gupta,[3,5] Seunghyun Weon,[2,3,6] Junfeng Niu,[4] Eli Stavitski,[7] Christopher Muhich,[3,5,*] and Jae-Hong Kim[2,3,*]

[1] Department of Environmental Science, Zhejiang University, Hangzhou, 310058, China
[2] Department of Chemical and Environmental Engineering, Yale University, New Haven, Connecticut 06511, USA
[3] NSF Nanosystems Engineering Research Center for Nanotechnology Enabled Water Treatment (NEWT)
[4] School of Environment and Civil Engineering, Dongguan University of Technology, Dongguan, Guangdong 523808, China
[5] School for the Engineering of Matter, Transport, and Energy, Arizona State University, Tempe, AZ 85281
[6] School of Health and Environmental Science, Korea University, Seoul 02841, Korea
[7] National Synchrotron Light Source-II, Brookhaven National Laboratory, Upton, NY 11973, USA

[#] These authors contributed equally
* Corresponding Author: Christopher.Muhich@asu.edu, jaehong.kim@yale.edu

Supplementary Note 1. Chemicals. All chemicals were used as received without further purification. Aminopropyltrimethoxysilane, 4-chlorophenol, 2,4-dichlorophenoxyacetic acid, 2,4,6-trichlorophenol, phenol, phenoxyacetic acid, isopropanol, and palladium(II) chloride were obtained from Sigma Aldrich. Acetonitrile (HPLC grade, >99.9%) was obtained from J. T. Baker. Silicon carbide (beta-phase, nanopowder, 95% purity) was obtained from Alfa Aesar. H_2 gas (ultrapure, 5.5 Grade) was obtained from Airgas. All solutions were prepared using ultrapure water (>18.2 MΩ•cm) produced by Millipore Milli-Q Water Purification System.

Supplementary Note 2. Analysis of 4-CP, 2,4-D, TCP, and their hydrodehalogenation products. Organohalides and their hydrodehalogenation products were quantified with an Agilent high-performance liquid chromatography (HPLC) coupled to a photodiode array (PDA) detector. Separation was carried out in a C18 column at 20 °C with a mobile phase of acetonitrile (A) and 0.1% phosphoric acid (B). Detailed analysis methods were listed in Table S3. Standard solution was used to calibrate the peak area in relation to parent compound and product concentrations.

Supplementary Note 3. Analysis of Cl^-. Cl^- was quantified with an ion chromatography (Dionex LC20; Sunnyvale, USA). Separation was carried out in a Dionex IonPac AG14A column (4 mm × 50 mm) at 25 °C with a mobile phase of 8.0 mM Na_2CO_3 and 1.0 mM $NaHCO_3$ at a flow rate of 1.0 mL/min. Sodium chloride standard solution was used to calibrate the peak area in relation to Cl^- concentration.

Supplementary Note 4. Calculation of turnover frequencies (TOF). The turnover frequencies (TOF) per Pd atom basis for 4-CP hydrodehalogenation was calculated based on the initial hydrogenation rate (R_d) of 4-CP at 0 min.

$$\mathrm{TOF} = \frac{R_\mathrm{d}}{C_\mathrm{Pd}} \quad (1)$$

For instance, the 4-CP hydrodehalogenation rate by 5.6%-Pd/SiC was 225 μM/min at 0 min. Therefore, the TOF by 5.6%-Pd/SiC was calculated as 225 μM/min / (0.5 g/L × 5.6%/106.4 g/mol) = 0.86 min^{-1}. The 4-CP hydrodehalogenation rate by Pd_{nano}/SiC was 3.5 μM/min at 0 min. Therefore, the TOF by Pd_{nano}/SiC was calculated as 3.5 μM/min / (0.5 g/L × 6.2%/106.4 g/mol) = 0.012 min^{-1}.

We also calculated the TOF of Pd_{nano} by taking into account of only surface atoms. The Pd dispersion of Pd_{nano}/SiC was calculated according to the following relationship between the dispersion (D) and particle radius (r): D/% = 100 × 5.6/r. The average particle radius of Pd_{nano}/SiC was 15 Å, corresponding to a dispersion of 37.3%. TOF of Pd_{nano}/SiC with surface atom normalization was 0.032 min^{-1}. In comparison, the dispersion for Pd single atom catalysts was 100%. The result suggests that, even after surface atom normalization, the TOF of 5.6%-Pd/SiC is still 27 times higher than that of Pd nanoparticle.

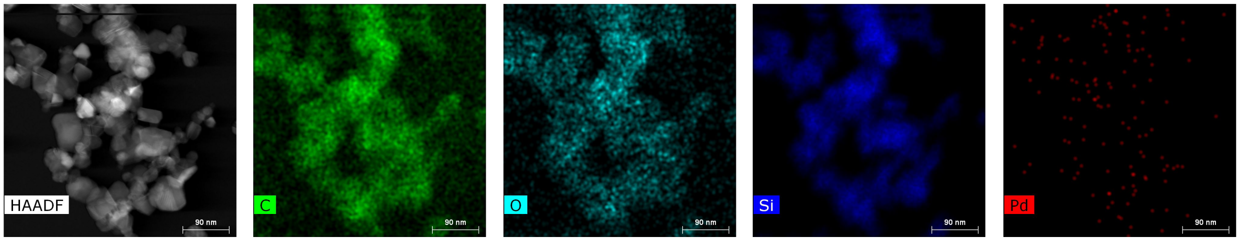

Supplementary Figure 1. EDS images of 5.6%-Pd/SiC.

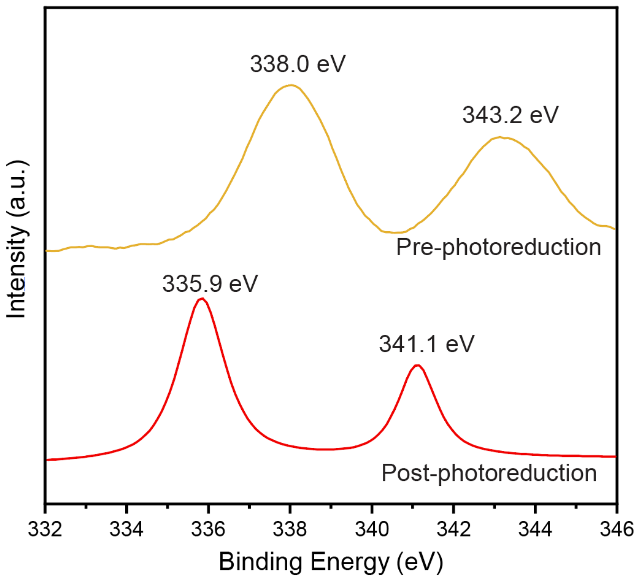

Supplementary Figure 2. Binding energy of Pd 3d for 5.6%-Pd/SiC before and after UV-C irradiation.

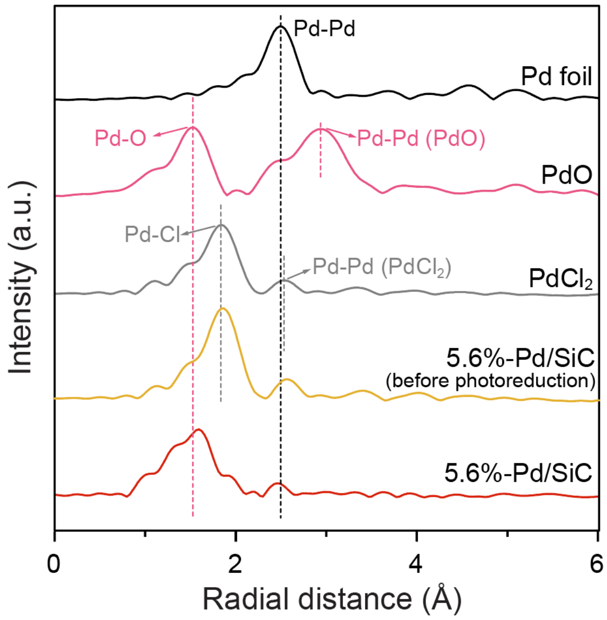

Supplementary Figure 3. Normalized Fourier-transform EXAFS spectra of 5.6%-Pd/SiC at the Pd K-edge before and after photoreduction.

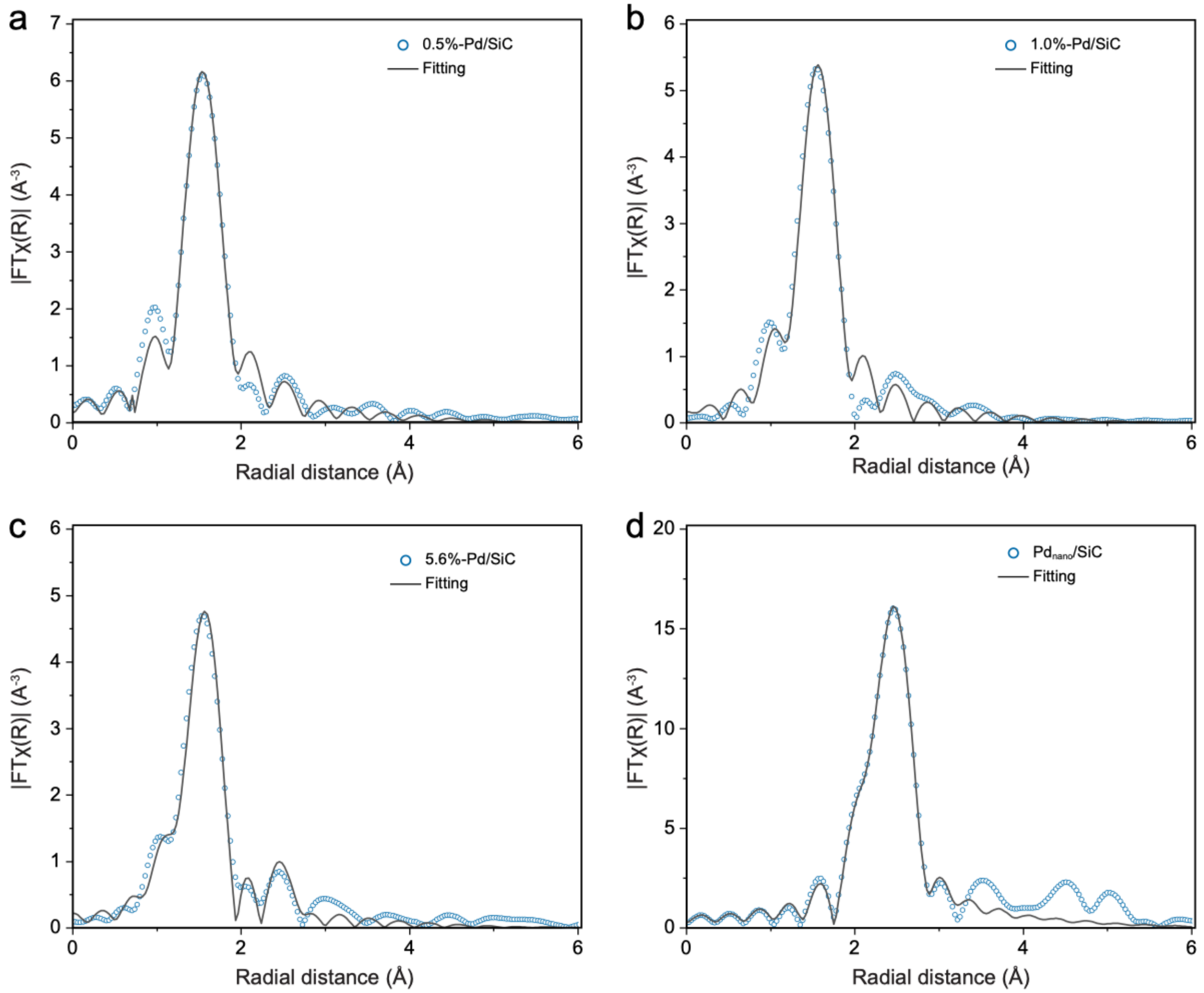

Supplementary Figure 4. Fits of 0.5%-Pd/SiC (a), 1.0%-Pd/SiC (b), 5.6%-Pd/SiC (c), and Pd_{nano}/SiC (d) EXAFS spectra.

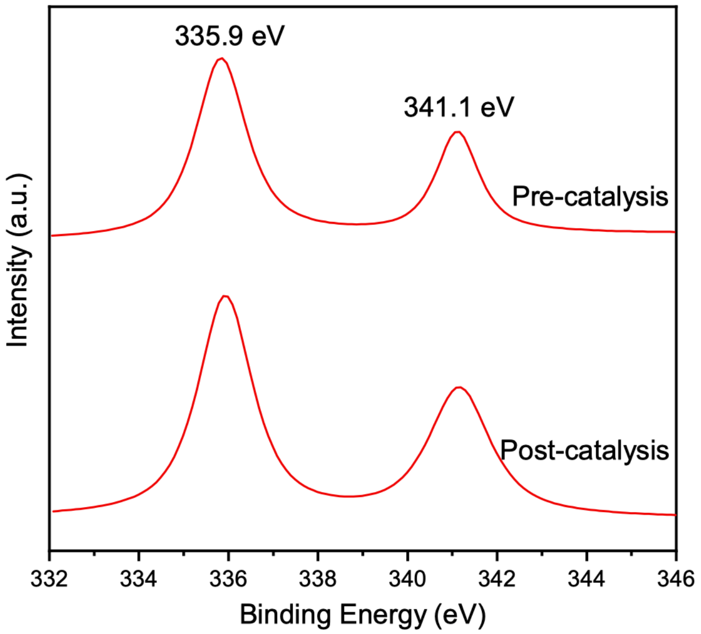

Supplementary Figure 5. Binding energy of Pd 3d for 5.6%-Pd/SiC before and after 1-week hydrogenation. Experimental conditions: catalyst (0.5 g/L), H_2 (1 atm), room temperature (20 °C).

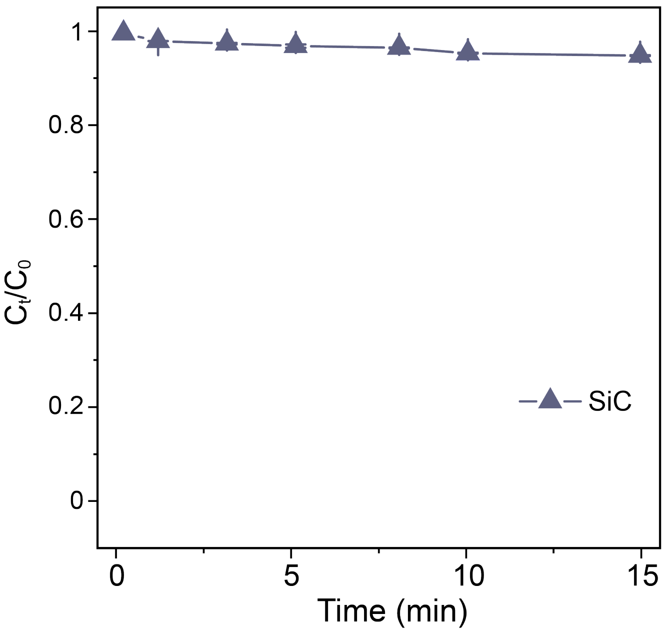

Supplementary Figure 6. Hydrodehalogenation kinetics of 4-CP by SiC. Experimental conditions: catalyst (0.5 g/L), H_2 (1 atm), room temperature (20 °C).

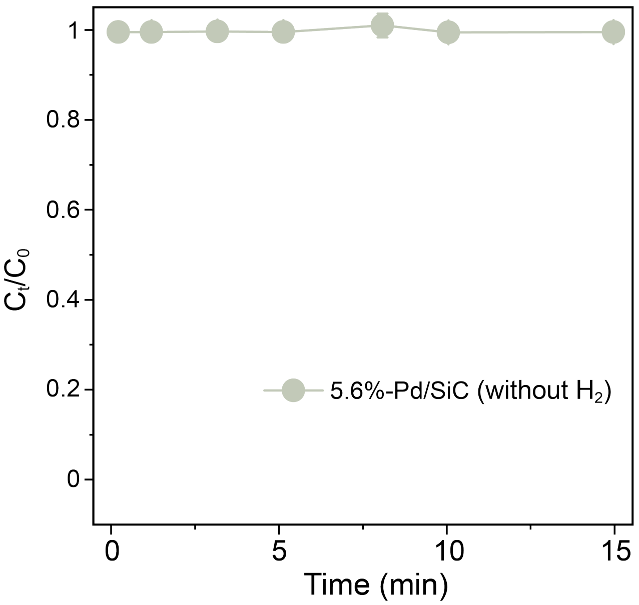

Supplementary Figure 7. Removal kinetics of 4-CP by 5.6%-Pd/SiC in the absence of H_2. Experimental conditions: catalyst (0.5 g/L), Air (1 atm), room temperature (20 °C).

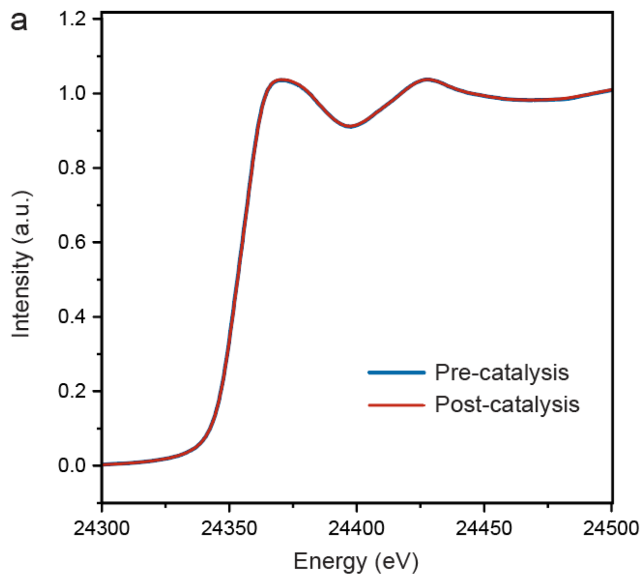

Supplementary Figure 8. Normalized XANES of 5.6%-Pd/SiC (a) and Fourier-transform EXAFS spectra of 5.6%-Pd/SiC at the Pd K-edge (b) before and after 1-week exposure under experimental conditions.

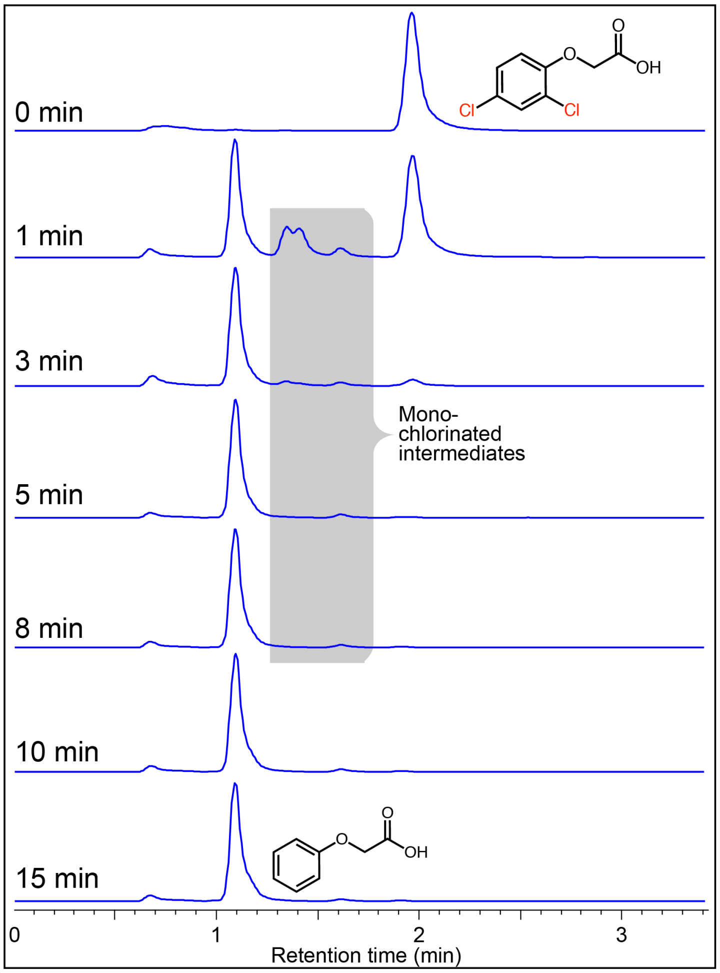

Supplementary Figure 9. HPLC absorption spectrum for 2,4-D hydrogenation and product generation.

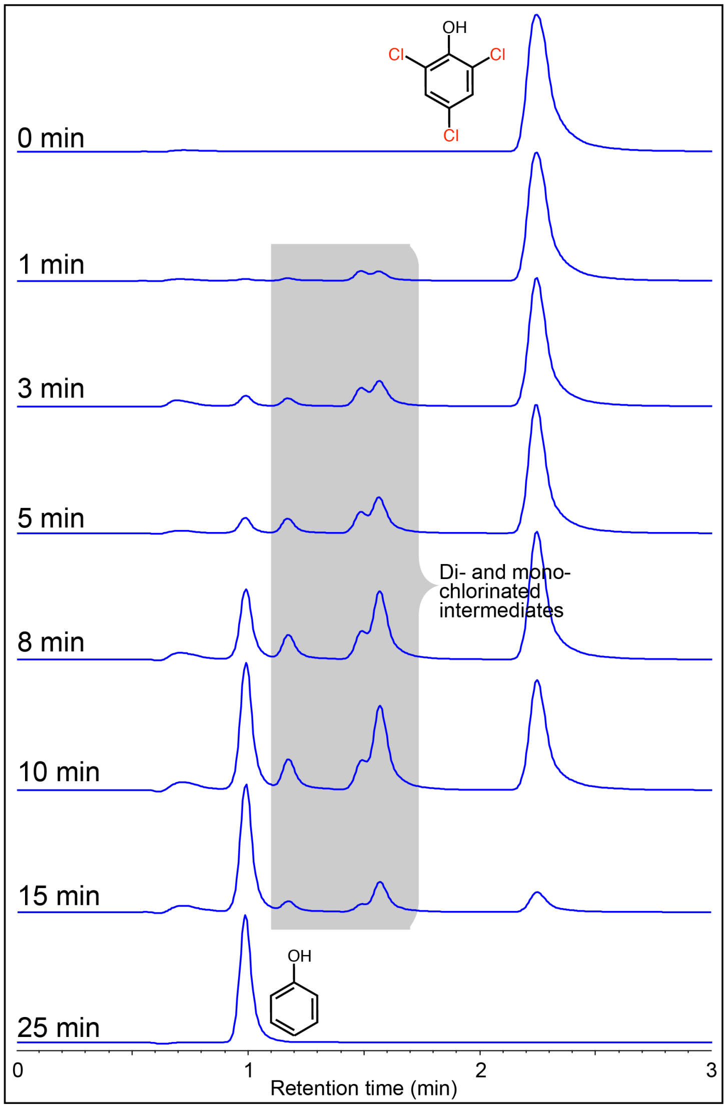

Supplementary Figure 10. HPLC absorption spectrum for TCP hydrogenation and product generation.

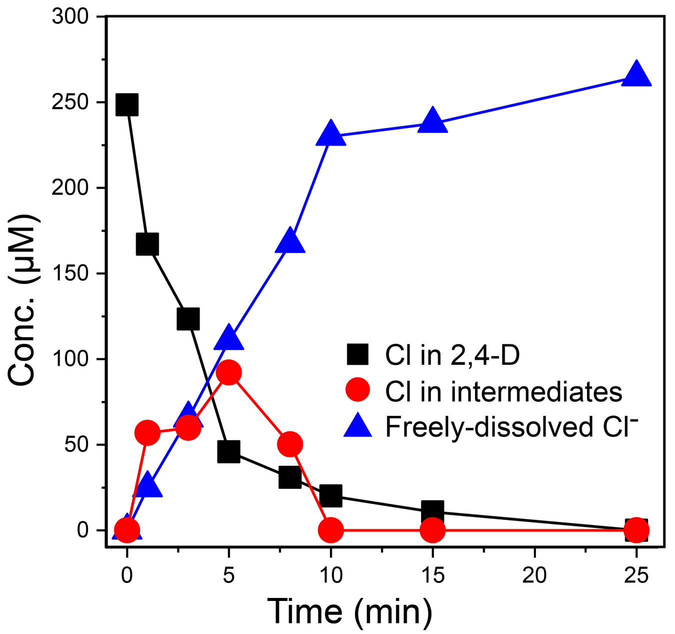

Supplementary Figure 11. Cl balance for hydrogenation of 2,4-D. Amount of Cl in intermediates was calculated by subtracting Cl in 2,4-D and freely-dissolved Cl^- from total Cl.

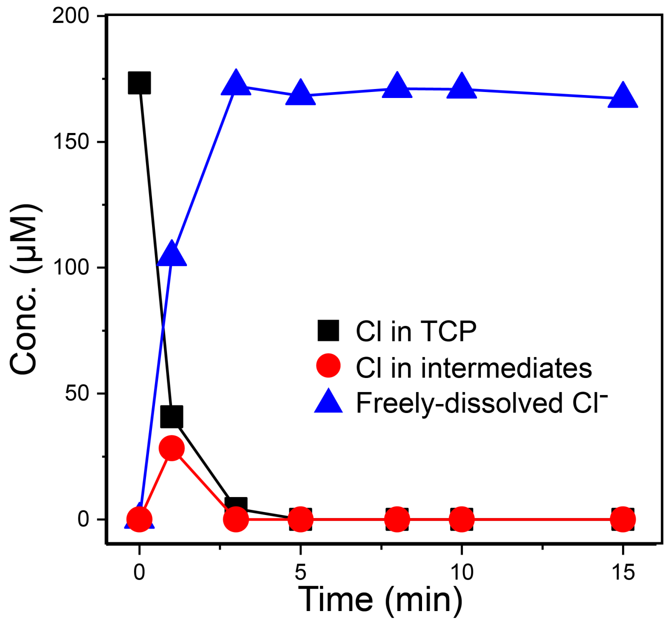

Supplementary Figure 12. Cl balance for hydrogenation of TCP. Amount of Cl in intermediates was calculated by subtracting Cl in TCP and freely-dissolved Cl^- from total Cl.

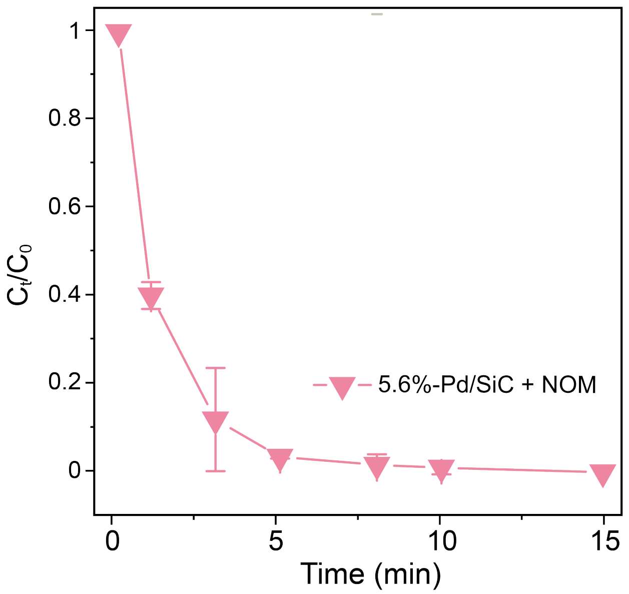

Supplementary Figure 13. Hydrodehalogenation kinetics of 4-CP by 5.6%-Pd/SiC in the presence of natural organic matter (Suwannee River NOM, Lot 2R101N, purchased from the International Humic Substances Society). Experimental conditions: catalyst (0.5 g/L), H_2 (1 atm), NOM (5 mg_C/L), room temperature (20 °C). Error bars represent standard deviations from triplicate experiments.

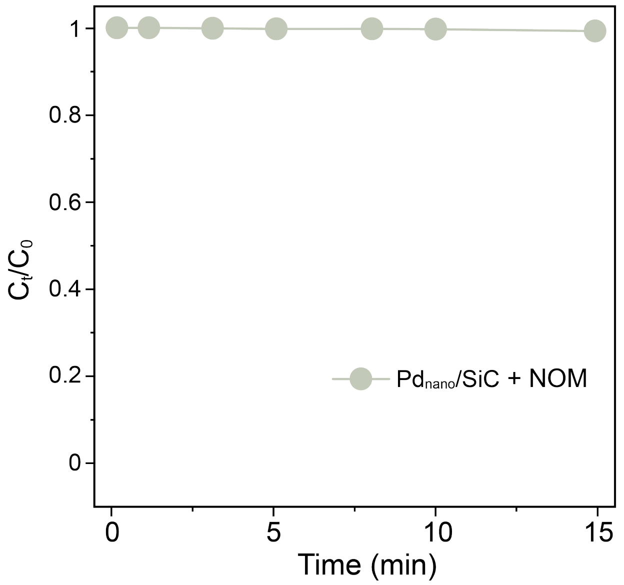

Supplementary Figure 14. Hydrodehalogenation kinetics of 4-CP by Pd$_{nano}$/SiC in the presence of natural organic matter (Suwannee River NOM, Lot 2R101N, purchased from the International Humic Substances Society). Experimental conditions: catalyst (0.5 g/L), H_2 (1 atm), NOM (5 mg$_C$/L), room temperature (20 °C).

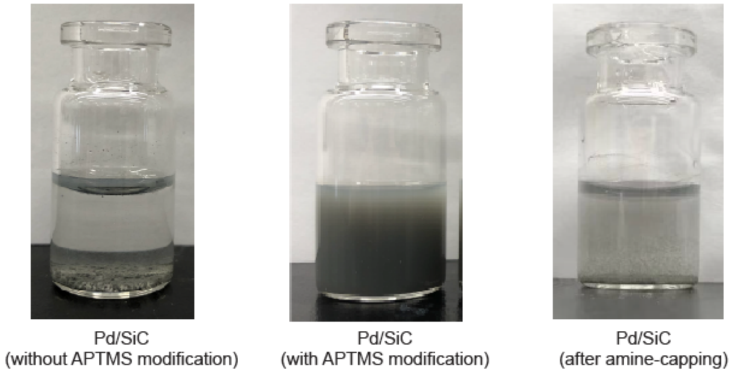

Supplementary Figure 15. Dispersion of 5.6%-Pd/SiC in deionized water.

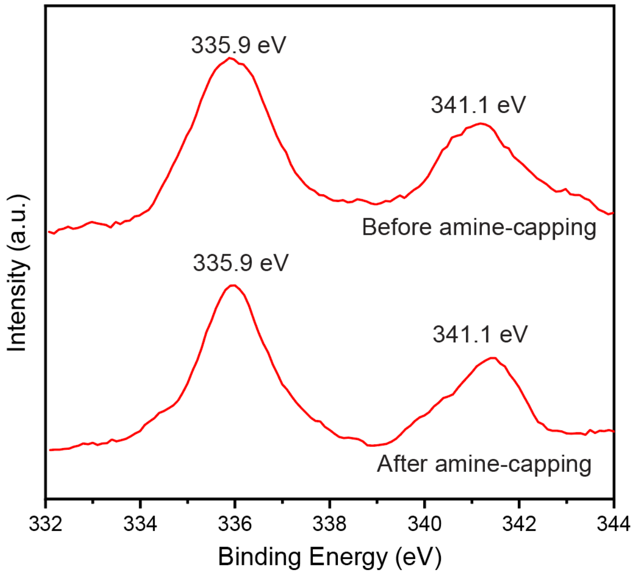

Supplementary Figure 16. Binding energy of Pd 3d for 5.6%-Pd/SiC before and after amine-capping.

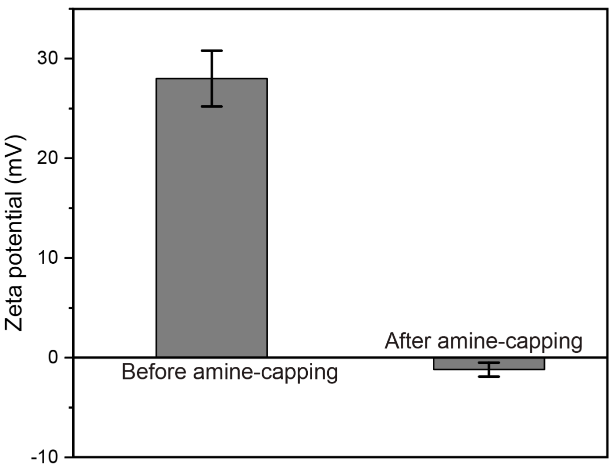

Supplementary Figure 17. Zeta potential of 5.6%-Pd/SiC before and after amine-capping. Amine-capping was achieved by mixing 20 mg 5.6%-Pd/SiC and 20 mg Sulfo-NHS-Acetate in 0.1 M sodium carbonate buffer (pH 8.5). For Zeta potential analysis, the samples were dried at 80 ˚C in an oven overnight, and dispersed in DI water before zeta potential measurements. The solution pH was around 7.0. Error bars represent standard deviations from thirty measurements.

Supplementary Table 1. Best fit parameters derived from fitting of Fourier-transform EXAFS spectra

Sample	Shell	Coordination number	r (Å)	$\Delta\sigma^2$ (Å^2)
0.5%-Pd/SiC	Pd-O	3.4 ± 0.4	2.03 ± 0.01	0.003 ± 0.002
1.0%-Pd/SiC	Pd-O	3.3 ± 0.4	2.05 ± 0.01	0.003 ± 0.001
5.6%-Pd/SiC	Pd-O	3.2 ± 0.3	2.04 ± 0.01	0.004 ± 0.001
	Pd-Pd	0.34 ± 0.08	2.74 ± 0.06	0.007 ± 0.018
Pd_{nano}/SiC	Pd-Pd	10.4 ± 0.5	2.75 ± 0.02	0.006 ± 0.000

Supplementary Table 2. Comparison of 4-CP removal kinetics.

Catalyst	Temperature	Pressure	Rate (h^{-1})	Selectivity	Ref.
Pd/active carbon	50 °C	2.4 bar	-	88%	1
Pd/active carbon	75 °C	2.4 bar	-	53%	1
Pd/active carbon	50 °C	2.4 bar	-	86%	1
Pd/active carbon	75 °C	2.4 bar	-	22%	1
Pd/Al_2O_3	20 °C	2 bar	0.45	91%	2
Pd/Al_2O_3	30 °C	2 bar	0.60	-	2
Pd/Al_2O_3	40 °C	2 bar	0.76	82%	2
Pt/Al_2O_3	20 °C	2 bar	0.26	55%	2
Pt/Al_2O_3	30 °C	2 bar	0.39	-	2
Pt/Al_2O_3	40 °C	2 bar	0.50	56%	2
Pt/Al_2O_3	20 °C	2 bar	0.64	20%	2
Pt/Al_2O_3	30 °C	2 bar	0.77	-	2
Pt/Al_2O_3	40 °C	2 bar	1.03	4%	2
Pd nanoparticle	30 °C	1 bar	1.56	-	3
Pd nanoparticle	30 °C	1 bar	0.66	-	3
Pd nanoparticle	30 °C	1 bar	4.3	-	3
Pd nanoparticle	30 °C	1 bar	1.56	-	3
Pd nanoparticle	30 °C	1 bar	3.48	-	3
AuPd	-	1 bar	15.6	-	4
AuAgPd	-	1 bar	16.5	-	4
Pd/C	-	1 bar	26.6	99%	5
PdRh	100 °C	27.6 bar	-	0.5%	6
Pd/Al_2O_3	-	4.1 bar	-	2.6%	7
Pd/Al_2O_3	30 °C	1 bar	21.2	75%	8
Rh/Al_2O_3	30 °C	1 bar	7.6	0%	8
0.5%-Pd/SiC	20 °C	1 bar	0.52	100%	This work
1.0%-Pd/SiC	20 °C	1 bar	2.4	100%	This work
5.6%-Pd/SiC	20 °C	1 bar	126	100%	This work
Pd_{nano}/SiC	20 °C	1 bar	2.1	66%	This work

Supplementary Table 3. HPLC analysis methods for organohalides and their hydrodehalogenation products.

Compounds		Acetonitrile (%)	Phosphoric acid (%)	Flow rate (mL/min)	Absorption (nm)	Retention time (min)
Parent	4-CP	45	55	2	225	2.00
Product	Phenol					1.32
Parent	2,4-D	50	50	2	225	1.96
Product	phenoxyacetic acid					1.09
Parent	TCP	60	40	2	205	2.24
Product	Phenol					0.99

Supplementary References

1. Calvo, L.; Gilarranz, M. A.; Casas, J. A.; Mohedano, A. F.; Rodríguez, J. J., Hydrodechlorination of 4-Chlorophenol in Water with Formic Acid Using a Pd/Activated Carbon Catalyst. *J. Hazard. Mater.* **161**, 842-847 (2009)
2. Díaz, E.; Casas, J. A.; Mohedano, Á. F.; Calvo, L.; Gilarranz, M. A.; Rodríguez, J. J., Kinetics of the Hydrodechlorination of 4-Chlorophenol in Water Using Pd, Pt, and Rh/Al2O3 Catalysts. *Ind. Eng. Chem. Res.* **47**, 3840-3846 (2008)
3. Baeza, J. A.; Calvo, L.; Gilarranz, M. A.; Mohedano, A. F.; Casas, J. A.; Rodriguez, J. J., Catalytic Behavior of Size-Controlled Palladium Nanoparticles in the Hydrodechlorination of 4-Chlorophenol in Aqueous Phase. *J. Catal.* **293**, 85-93 (2012)
4. Liu, R. et al. Au@Pd Bimetallic Nanocatalyst for Carbon-Halogen Bond Cleavage: An Old Story with New Insight into How the Activity of Pd Is Influenced by Au. *Environ. Sci. Technol.* **52**, 4244-4255 (2018)
5. Dong, Z.; Dong, C.; Liu, Y.; Le, X.; Jin, Z.; Ma, J., Hydrodechlorination and Further Hydrogenation of 4-Chlorophenol to Cyclohexanone in Water over Pd Nanoparticles Modified N-Doped Mesoporous Carbon Microspheres. *Chem. Eng. J.* **270**, 215-222 (2015)
6. Bovkun, T. T.; Sasson, Y.; Blum, J., Conversion of Chlorophenols into Cyclohexane by a Recyclable Pd-Rh Catalyst. *J. Mol. Catal. A-Chem.* **242**, 68-73 (2005)
7. Roy, H. M.; Wai, C. M.; Yuan, T.; Kim, J.-K.; Marshall, W. D., Catalytic Hyrodechlorination of Chlorophenols in Aqueous Solution under Mild Conditions. *Appl. Catal. A-Gen.* **271**, 137-143 (2004)
8. Munoz, M.; de Pedro, Z. M.; Casas, J. A.; Rodriguez, J. J., Improved Γ-Alumina-Supported Pd and Rh Catalysts for Hydrodechlorination of Chlorophenols. *Appl. Catal. A-Gen.* **488**, 78-85 (2014)
